# Adolescent social defeat decreases spatial working memory performance in adulthood

**DOI:** 10.1186/1744-9081-9-39

**Published:** 2013-10-17

**Authors:** Andrew M Novick, Leah C Miiller, Gina L Forster, Michael J Watt

**Affiliations:** 1 Center for Brain and Behavior Research, Division of Basic Biomedical Sciences, Sanford School of Medicine, University of South Dakota, 414 East Clark Street, Vermillion, SD 57069-2390, USA

**Keywords:** Adolescence, Stress, Social defeat, Spatial working memory, Dopamine, Prefrontal cortex

## Abstract

**Background:**

Adolescent social stress is associated with increased incidence of mental illnesses in adulthood that are characterized by deficits in cognitive focus and flexibility. Such enhanced vulnerability may be due to psychosocial stress-induced disruption of the developing mesocortical dopamine system, which plays a fundamental role in facilitating complex cognitive processes such as spatial working memory. Adolescent rats exposed to repeated social defeat as a model of social stress develop dopaminergic hypofunction in the medial prefrontal cortex as adults. To evaluate a direct link between adolescent social stress and later deficits in cognitive function, the present study tested the effects of adolescent social defeat on two separate tests of spatial working memory performance.

**Methods:**

Adult rats exposed to adolescent social defeat and their controls were trained on either the delayed win-shift task or the delayed alternating T-Maze task and then challenged with various delay periods. To evaluate potential differences in motivation for the food reward used in memory tasks, consumption and conditioned place preference for sweetened condensed milk were tested in a separate cohort of previously defeated rats and controls.

**Results:**

Compared to controls, adult rats defeated in adolescence showed a delay-dependent deficit in spatial working memory performance, committing more errors at a 90 s and 5 min delay period on the T-maze and win-shift tasks, respectively. Observed memory deficits were likely independent of differences in reward motivation, as conditioned place preference for the palatable food used on both tasks was similar between the adolescent social defeat group and control.

**Conclusions:**

The results demonstrate that severe social stressors during adolescence can produce long term deficits in aspects of cognitive function. Given the dependence of spatial working memory on prefrontal dopamine, pharmacologically reversing dopaminergic deficiencies caused by adolescent social stress has the potential to treat such cognitive deficits.

## Background

The experience of social stressors such as bullying during adolescence places individuals at high risk for developing various psychiatric disorders both acutely and later in life, and include depression [1,2], anxiety [1,3], attention deficit hyperactivity disorder (ADHD) [4,5], and substance abuse [6,7]. While these disorders can present with widely varying symptoms, each is characterized by deficits in executive function [8], thus providing a common variable by which to study the consequences of adolescent stress across a range of separate diagnoses. Broadly defined, executive function encompasses cognitive processes that allow for organized and adaptive behavior, including the ability to utilize and maintain task-relevant information, otherwise known as spatial working memory [9]. Of particular relevance to adolescent social stress, both executive function ability and its major associated brain region, the prefrontal cortex (PFC), continue to mature during the adolescent period [10]. The maturation of the adolescent PFC is delayed compared to both subcortical structures [11] and to other cortical regions [12,13]. This appears to render the adolescent PFC susceptible to long-lasting neural disruption from social stressors [14], which may contribute to the cognitive deficits seen in stress-associated disorders. The susceptibility of the developing PFC to stress is reflected by the finding of one pilot study that emotional abuse during childhood is associated with decreases in human adult spatial working memory [15]. Furthermore, structural variations in the PFC appear to mediate the relationship between early life stressors and later spatial working memory deficits [16], suggesting that early life stressors may alter the structure of the developing PFC to contribute to cognitive dysfunction later in life. While such studies provide important retrospective data on the potential consequences of early life stressors on cognitive ability, the direct effects of adolescent social stress on executive function and its underlying neural mechanisms have yet to be explored.

We have developed a paradigm using social defeat of adolescent rats to model the victimization and imbalance of power inherent in human social stress experiences, including teenage bully-victim relationships [17,18]. Following exposure to adolescent social defeat, several significant behavioral and neural alterations emerge in young adulthood [18-21]. In particular, rats defeated in adolescence show decreased dopamine (DA) content in the medial PFC (mPFC) in adulthood at baseline [18] and blunted extracellular mPFC DA release when challenged with acute amphetamine [19,22]. Adolescent defeat also results in increased expression of DA transporters in the adult mPFC [21], which our preliminary data suggest enhances DA clearance and contributes to lowered mPFC DA activity [23]. In addition, previously defeated rats show several behaviors indicative of mPFC DA hypofunction including increased locomotor responses to novel environments [18,19] as well as increased conditioned place preference for amphetamine [20]. These results are noteworthy given that mesocortical DA plays a critical role in mediating executive function [9]. Depletions of mPFC DA are known to produce deficits in spatial working memory tasks in both primates and rats [24-26], and this appears to modulated by a lack of dopaminergic activity at the DA D1 receptor [27,28]. However, the relationship between spatial working memory performance and DA D1 stimulation appears to be based on an inverted-U function, as performance deficits are noted following both D1 agonism [29] and blockade [28]. Importantly, mPFC DA release decreases as the task delay is prolonged, and this decay in release correlates with increased spatial working memory errors [30]. Pharmacological D1 stimulation can rescue memory performance during either long delays or following poor baseline performance, but is detrimental during shorter delays or when baseline performance is already high [29,31-33]. These studies suggest that an optimal level of mPFC DA activity is needed to maintain and utilize task-specific information across varying delay periods.

Previous research has demonstrated that stress in the form of water restraint [32] or benzodiazepine antagonism [34] in adult animals leads to acute disruptions to DA-dependent spatial working memory. While evidence suggests that adolescent social stressors may result in long term disruption to hippocampal-dependent spatial memory [35-37], we are currently unaware of any research that has tested protracted changes in mPFC DA-dependent spatial working memory as a result of stress in adolescence. Given our previous findings of mPFC DA hypofunction following adolescent social defeat, we hypothesized that previously defeated rats would demonstrate disruptions in spatial working memory performance in adulthood. Therefore, we employed two separate spatial working memory tasks that differ in terms of delay period used. In the delayed win-shift task, rats acquire spatial information that then must be utilized to guide search behavior across delays that typically range from 5 minutes to 6 hours [34]. The second task, the delayed alternating T-maze, also requires memory to guide search behavior but commonly utilizes shorter delays of 0 to 90s [26]. Furthermore, because these tasks use food reward (sweetened condensed milk [SCM]), baseline SCM consumption and conditioned place preference for SCM were tested following adolescent social defeat to rule out potential differences in task motivation that could confound results of the spatial working memory tests [38,39]. As predicted, adolescent social defeat caused impairments in both spatial working memory paradigms, which were dependent on the delay period used. Moreover, these deficits appear to be independent of differences in motivation for the food reward used in the memory tasks.

## Methods

### Animals

Male juvenile post-weanling Sprague–Dawley rats (Postnatal day [P]21, n = 72) were obtained from the University of South Dakota Laboratory Animal Services. All rats were pair-housed such that cage-mates were in the same treatment group (social defeat or control) and kept at 22°C on a reverse 12-hr light–dark cycle (lights off 10.00). Animals were pair-housed for the entire experiment to avoid the confound of social isolation or social disruption on the developing brain and behavior [35,40]. Food and water were available *ad libitum*, until P56. To facilitate assessment of spatial working memory, at P56, rats underwent mild food restriction to 85% of their free feeding weight [26,34] with 5 g per week body weight increase allowed to accommodate for normal growth. Behavioral experiments were conducted under red lighting during the dark-phase of the light cycle, between 11.00 and 18.00. All procedures were carried out in accordance with the United States National Institutes of Health Guide for the Care and Use of Laboratory Animals and received approval from the Institutional Animal Care and Use Committee of the University of South Dakota. Every effort was made to minimize the number of animals used and their suffering.

### Adolescent social defeat

The adolescent social defeat procedure used in the present study was conducted as described previously [18,19]. At P35 (mid-adolescence [41,42]), 36 male rats were placed separately in the cage of a larger, aggressive adult Sprague Dawley resident male rat once daily for 5 consecutive days. Social defeat was defined *a priori* as the adolescent intruder adopting 3 consecutive submissive supine postures in response to resident attacks [18], which typically occurs within 5 min. Immediately following 10 min exposure to social defeat, the adolescent and resident were separated by a wire mesh barrier for an additional 25 min to prevent further physical attack while still allowing exposure to visual, auditory, and olfactory intimidation from the resident [18,19]. Pairs of adolescent rats were removed from their home cage and exposed to social defeat simultaneously, and were returned together to their home cage immediately after each daily trial. Pairs of aged-matched controls (n = 36) did not undergo social defeat but were instead placed simultaneously into separate novel empty cages for the duration of the defeat procedure to control for handling and novel environment stress [18], and then returned together to their home cage. After the final defeat trial, all rats were allowed to mature undisturbed in pairs according to treatment group (defeat or control) in their home cages until early adulthood (P56). It should be noted that defeat experience may negatively alter subsequent social interaction within the home cage, which could compound the effects of defeat alone on later non-social behaviors such as spatial working memory. We did not include comparison groups of defeated rats and controls with no further opportunity for home cage interaction (i.e., social isolates) in the current study to investigate this possibility, given the known detrimental effects of social isolation on later behavior especially when experienced during adolescence [35,40].

### Experimental overview

Following adolescent social defeat and a brief period of food restriction (see below), training for spatial working memory tests commenced at approximately P60 using two separate spatially based paradigms, the delayed win-shift task [34] and the delayed alternating T-maze task [26], the details of which are described below. Depending on individual training progress, spatial working memory assessment was started approximately between P65-P70 (young adulthood, ~4 weeks following completion of social defeat procedure). Separate cohorts of rats were used for each task. An additional separate cohort was used to evaluate consumption and conditioned place preference for the reward used in both spatial working memory tasks (sweetened condensed milk; SCM) to rule out differences in reward motivation that might affect spatial working memory task performance [38,39].

### Food restriction

At P56, all rats underwent food restriction to 85% of body weight in order to facilitate food conditioning for food reward-based spatial working memory assessment [26,34]. Cagemates were separated for 1–2 hours each day by a wire mesh barrier and each was given approximately 10 g of standard rat chow along with a water dish. Target body weight was achieved in 5–6 days. To accommodate growth, the target body weight was increased by 5 g each week. Rats were weighed daily prior to feeding and provided with 10–20 g of standard lab chow in order to maintain target weight. Water was always available outside feeding times. Following completion of experimental testing, rats were returned to *ad libitum* feeding.

### Conditioned place preference (CPP) for food reward

The sweetened condensed milk (SCM) CPP protocol utilized a biased design based on that of Schneider et al. [43]. After food restriction, rats (social defeat n = 12, control n = 12) were acclimated to the CPP apparatus (Med Associates, Inc., St Albans, CT, USA) consisting of two conditioning chambers (21 cm wide × 21 cm high × 28 cm long) separated by a small center chamber (21 cm wide × 21 cm high × 12 cm long). The conditioning chambers differed by both color (white vs. black) and floor type (steel mesh vs. rod). Acclimation consisted of 3 × 30 minute sessions in which the rat was allowed to freely explore the apparatus, with movement and time spent in each chamber measured by photobeam breaks. Following the first two acclimation sessions, rats were returned to their cage and given a small dish of SCM diluted in tap water (1:3, [43]) in order to facilitate habituation to the novel food. After the third acclimation session, rats were separated in their home cage for 30 minutes by a mesh barrier and each given a water bottle filled with dilute SCM in order to assess consumption in a familiar environment (g consumed over the 30 min period).

All rats exhibited a pre-preference for the black chamber across the three acclimation sessions. For this reason, the subsequent conditioning trials involved the white side of the apparatus always being paired with SCM (free access to a bottle of diluted SCM) and the black side of the apparatus always being paired with tap water. The day after the final acclimation, rats underwent four 30 min sessions of conditioning across two days which involved each rat being exposed to both chambers on each day with a four hour period between each conditioning session within a day, and a reversed order of chamber exposure on the second day. The amount of SCM consumed during each conditioning session was recorded. On the test day, bottles were removed from the apparatus and rats were placed in the center chamber and given access to all compartments for 30 mins. The CPP ratio was calculated as the time spent on the test day in the SCM-paired chamber divided by the time spent in the non-SCM chamber [43].

### ***Delayed win-shift task***

The method for the delayed win-shift task was based on that used by Floresco et al. [34] and Andersen et al. [44] with minor modifications. An 8-arm radial arm maze (Noldus Information Technology, Leesburg VA) was utilized, consisting of a center platform (24 cm in diameter) connected to 8 individual walled arms (60 cm long × 10 cm wide × 35 cm high) with cylindrical food cups at the end of each arm. Access to individual arms was controlled by mechanical guillotine doors that were operated by Noldus Ethovision 3.1 (Noldus Information Technology). Rats (a separate cohort from those that underwent CPP testing, n = 12 per social defeat and control group) underwent habituation to the maze followed by training without a delay, and finally testing with delays. During habituation, rats were given free access to explore the entire maze for 15 minutes on two consecutive days. On the first day of habituation, drops of sweetened condensed milk (SCM, diluted 1:1 with water, [45]) were scattered throughout the entire maze with three drops in each food cup. On the second day of habituation, only the food cups contained SCM. Following each habituation exposure, the rats were returned to their cages and given a small dish containing 16 drops of SCM in addition to their daily food ration in order to promote further acclimation to the novel food [46].

Training trials for the delayed win-shift task consisted of two phases. In the *acquisition* phase, the rat was placed in the center of the maze with all doors closed. All arms were baited with 1 drop of SCM in each terminal food cup. The rat was then given access to 4 randomly selected arms. An arm entry was recorded when the rat reached the food cup at the end of an arm. Once the rat had visited each of the 4 arms, all doors were closed and the rat was returned immediately to the center of the maze to start the *retrieval* phase of the task. In this phase, all doors were opened, but SCM was only located in the arms that the rat had not visited in the previous acquisition phase. An error was recorded in the retrieval phase if the rat visited one of the baited arms twice or visited one of the non-baited arms. After the rat entered all the baited arms, it was returned to its cage. If a rat failed to enter all 4 baited arms within 5 min in either the acquisition or retrieval phase, the training session was terminated and the rat was returned to its cage. The maze was wiped down with dilute vinegar and water between subjects. Rats were trained in this manner twice daily until they reached a performance criterion of 1 error or less during the retrieval phase for two consecutive training sessions. Upon reaching criterion, each rat was then tested once daily with a 5, 30, 60, and 480 min delay in between the acquisition and retrieval phases across consecutive days. Once the rat consumed all four rewards in the acquisition phase, all doors were closed and the rat was placed in its home cage for the duration of the delay. Before returning to the center of the maze for the retrieval phase, the maze was wiped down with vinegar and water to remove olfactory cues. As with training, arm entries and errors were recorded by the experimenter.

### ***Delayed alternating T-maze task***

The protocol for the delayed alternating T-Maze task was adapted from Clinton et al. [26]. The T-Maze task consisted of habituation trials, followed consecutively by alternating shaping trials, alternation training without a delay, and then delayed alternation testing. Access to the opposing arms of the T-maze apparatus (69 cm long × 15 cm wide × 30 cm high) was controlled by removable guillotine doors located at the entry of each arm, with another removable door blocking the distal third of central arm (50 cm long × 15 cm wide × 30 cm high) to create a start box (30 cm long × 15 cm wide × 30 cm high). Rats (a separate cohort from those that underwent either CPP or delayed win-shift testing, n = 12 per social defeat and control group) were habituated to the entire T-Maze apparatus for 5 min twice a day for two consecutive days. On the first day, drops of diluted (1:1, [45]) SCM were scattered throughout the maze as well as on a small elevated platform at the end of each opposing arm. On the second day, only three drops of diluted SCM were placed on each platform. After each habituation session, rats were returned to their home cages and given a small dish containing 30 drops of diluted SCM in addition to their daily food ration in order to further acclimate the rats to the novel food. Following habituation, all rats were underwent alternation shaping, in which the rat was placed in the start box and one opposing arm of the T-Maze blocked. After the rat consumed the SCM from the open arm, this arm was blocked and the rat was returned to the start box for a second run in which access was allowed into the previously blocked but now baited arm, forcing the rat to alternate direction from the previous run in order to obtain the reward. Alternation shaping took place over two days, with each rat completing 10 alternation runs in both the morning and afternoon. Alternation training without a delay consisted of an *information run* and a *choice run*. For the *information run*, the rat was placed in the start box with one randomly selected arm blocked and the other baited with one drop of SCM. After the rat reached the terminal platform and consumed the milk, it was immediately returned to the start box for the beginning of the *choice run*. During the *choice run*, the rat had access to both arms of the T-Maze, but only the arm that was previously blocked in the information run was baited with SCM. An arm entry was recorded when the rat reached the terminal platform at the end of the arm. Regardless of which arm the rat entered on the choice run*,* it was subsequently returned to the start box for the beginning of the next information run. Each rat completed 10 of these training trials per session, and was given two sessions a day until reaching a criterion of 80% correct alternations for two consecutive sessions. Once a rat reached criterion, it began delayed alternation testing the following morning. Delayed alternation testing was identical to training, with the exception that a 30, 60, or 90 s delay was interposed in between the information run and the choice run. During the delay period, rats were placed in an empty cage covered with a sheet. For four consecutive days, rats were given one test session per day, which consisted of nine delay trials (three trials of each delay period). The order in which different delay periods were presented was randomized for each test day. Thus, while rats reached criterion and started testing at their own pace, each received the same random order of delay periods during testing.

### Data analysis

#### SCM consumption and conditioned place preference

Rats that failed to show SCM CPP (defeats, n = 4, controls, n = 4) were not included in any of the analyses assessing SCM consumption and CPP [20,47]. Homecage consumption (total g in 30 mins) of SCM following the final CPP acclimation session was corrected against individual body weight (kg) and then compared between defeated rats and controls using a one-way ANOVA in which significant main effects were further analyzed by post-hoc tests of Least Significant Difference (LSD) . Weight-corrected SCM consumption across the CPP conditioning sessions was compared using a two-way repeated measure ANOVA (adolescent stress [social defeat or control] × repeated factor of conditioning session). A significant effect of conditioning session was followed by one-way ANOVA with repeated measures and post-hoc LSD tests, while significant interactions between stress and conditioning session were further analyzed with LSD pairwise tests. To assess potential differences in place preference conditioning, one-way ANOVA with LSD tests were used to compare the ratio of time spent in the SCM chamber vs. the non-SCM chamber.

### Spatial working memory

Grubbs’ outlier tests to remove significant outliers were performed prior to the below analyses [48]. This resulted in the removal of data from one control animal in the delayed win-shift task for the 5 min delay period. For the delayed T-Maze task, separate Grubbs’ tests were applied to individual animal performance across test sessions for each delay period, resulting in the removal of 25 data points (9% of total). Furthermore, one animal in the social defeat group failed to reach criterion following 12 training sessions and was thus excluded from all subsequent T-maze testing and analyses. The number of trials to reach criterion for both the delayed win-shift and delayed-alternating T-maze tasks were compared using separate one-way ANOVA followed by LSD tests for multiple comparisons. Errors in the delayed win-shift task were compared using a two-way ANOVA with repeated measures (adolescent stress × delay period). One-way repeated measures ANOVA was performed when a significant effect of delay period was observed followed by LSD tests where appropriate. *Post-hoc* analysis of significant main effect of stress or an interaction was completed using pair-wise LSD tests. The fraction of correct alternations during the delayed alternating T-Maze were collapsed across test sessions and compared using a two-way repeated measures ANOVA (adolescent stress × delay period), with a significant stress × delay period interaction analyzed by pairwise LSD tests. All statistical tests were performed using SPSS 10.0, with the alpha level set *a priori* to 0.05.

## Results

### SCM consumption and conditioned place preference

**Figure 1:**
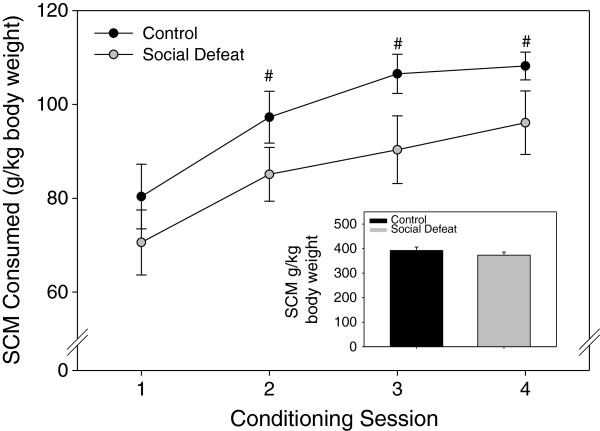
**Effect of adolescent social defeat on adult consumption of sweetened condensed milk (SCM) during CPP conditioning.** Inset: Total SCM consumed during all conditioning sessions. All data are expressed as mean ± SEM, n = 8 per group. #Significantly different (p < 0.05) within group from consumption on Day 1.

**Figure 2:**
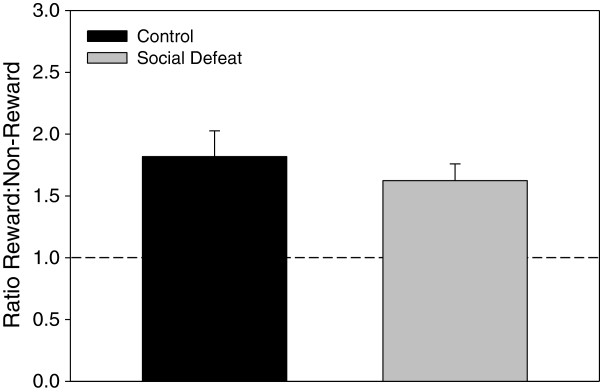
**Adolescent social defeat does not alter CPP for SCM in adulthood.** Data are expressed as the mean ± SEM of the ratio between time spent in the SCM-paired compartment to time spent in the non-SCM compartment, n = 8 per group. Means above the dashed line indicate place preference conditioning.

Both defeated and control rats were found to consume similar amounts of SCM across acclimation and conditioning sessions. Specifically, when SCM was presented in the homecage of rats following the third acclimation session to the CPP apparatus, there were no significant differences in amount consumed between rats that underwent adolescent social defeat and controls (F(1,14) = 0.202, p = 0.660 (data not shown). During the actual CPP conditioning sessions, comparison of SCM consumption revealed a significant main effect of both stress treatment (F(1,14) = 7.485, p < 0.05), and session (F(3,14) = 8.426, p < 0.001), but no interaction between these factors (F(3,42) = 0.107, p = 0.956). Within the control group, one-way repeated measures ANOVA revealed a significant effect of conditioning session on SCM consumption (F(3,21) = 9.050, p < 0.001), with SCM consumption during session two (LSD p < 0.05), session three (LSD p < 0.005) and session four (LSD p < 0.001) significantly greater than on session 1 (Figure 1). The social defeat group exhibited a weak trend towards similarly increasing consumption over time, but this effect was not significant (F(3,21) = 2.481, p = 0.089; Figure 1), However, the total amount of SCM consumed across conditioning was equivalent between previously defeated and control rats (F(1,14) = 1.023, p = 0.329; Figure 1 inset). In terms of preference for cues associated with SCM reward in adulthood, analysis of the CPP test session following conditioning revealed that the ratio of time spent in the SCM-paired compartment compared to the non-SCM compartment did not differ between rats defeated in adolescence and control groups (F(1,14) = 0.610, p = 0.448; Figure 2).

### Spatial working memory tasks

#### Delayed win-shift task

**Figure 3:**
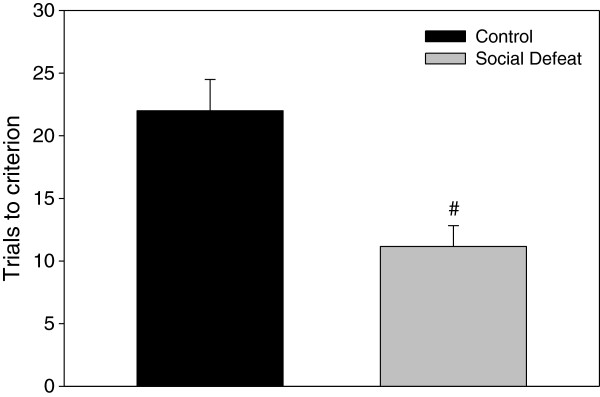
**Rats defeated in adolescence require less training trials than controls to reach criterion on the delayed win-shift task.** Data are expressed as the mean ± SEM, n = 12 per group. #Significant difference (p < 0.05) compared to control group.

**Figure 4:**
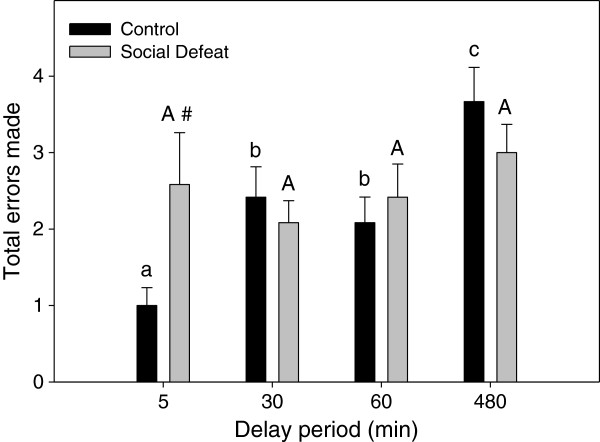
**Adolescent social defeat reduces spatial working memory performance in adulthood on the delayed-win shift task performance when 5 min delays are used.** Data are expressed as the mean ± SEM number of total errors committed during the retrieval phase of the task, n = 12 per group. #Significant difference (p < 0.05) compared to controls within 5 min delay period. For across delay period comparisons, means that do not share the same letter are significantly different (p < 0.05) (upper case letters = comparisons across delay periods for social defeated rats; lower case letters = comparisons across delay periods for controls).

During task training without a delay, rats that underwent adolescent social defeat required significantly fewer trials to reach the performance criterion of one error or less for two consecutive trials (F(1,22) = 13.088, p < 0.005; Figure 3). When total errors were compared during testing across delay times, there was a significant main effect of delay period (F(3,63) = 5.222, p < 0.005), and a significant interaction between adolescent stress and delay period (F(3,63) = 3.098, p < 0.05), but no main effect of adolescent stress (F(1,21) = 0.294, p = 0.594). *Post-hoc* analysis revealed that within the 5 min delay test, defeated rats made significantly more total errors compared to controls (Figure 4; LSD p < 0.05). However, performance for the 30, 60, and 480 min delay trials was similar between treatment groups (Figure 4; LSD lowest p = 0.307). One-way repeated measures ANOVA revealed that control animals demonstrated an expected increase in errors as the delay period increased (F(3,30) = 10.625, p < 0.001; Figure 4), with errors following both the 30 min and 60 min delays being significantly increased compared to the 5 min delay condition (Figure 4; LSD p < 0.05) and errors made following the 480 min delay significantly greater than all other delays (Figure 4; LSD highest p < 0.05). In contrast, errors committed within the defeated group were similar across all delay periods (F(3,33) = 0.737, p = 0.537; Figure 4).

#### Delayed alternating T-maze

**Figure 5:**
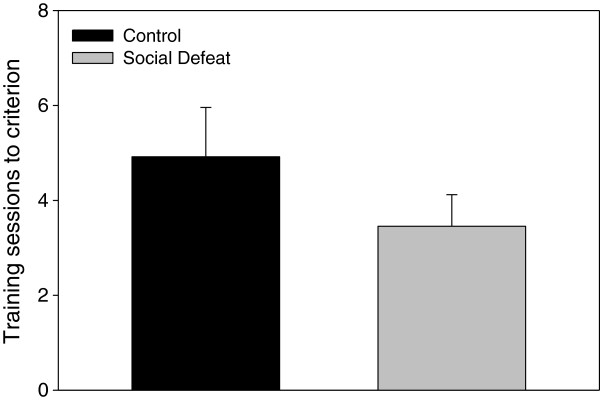
**Training trials needed to reach criterion on the delayed alternating T-maze in rats subjected to adolescent social defeat and their controls.** Data are expressed as the mean ± SEM for each group (controls n = 12, social defeat n = 11).

**Figure 6:**
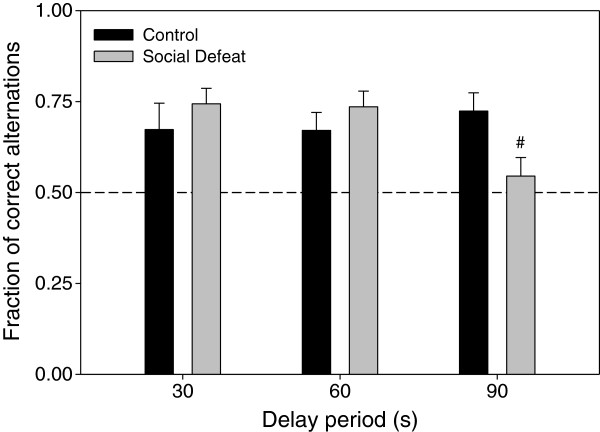
**Rats defeated in adolescence make fewer correct alternations on the delayed alternating T-maze task as adults with delay periods of 90 sec.** Data are expressed as the mean ± SEM of the fraction of correct alternations made. The dashed line represents the chance level of correct alternations being made. #Significant difference (p < 0.05) compared to controls within the 90s delay period (control n = 12, social defeat n = 11).

For alternation training on the T-maze, there were no significant differences in the number of training sessions needed to reach criterion between defeated rats and controls (F(1,21) = 1.344, p = 0.259; Figure 5). Two-way repeated measures ANOVA to assess alternation performance (Figure 6) yielded no main effects of either adolescent stress (F(1,21 ) = 0.118, p = 0.735) or delay period (F(2,42) = 1.165, p = 0.322), but there was a significant interaction between adolescent stress and delay period (F(2,42) = 3.456, p < 0.05). *Post-hoc* analysis found that the fraction of correct alternations in the social defeat group was significantly lower than control animals during trials with a 90 s delay (Figure 6; LSD p < 0.05).

## Discussion

The results of the present study demonstrate that exposure to repeated social defeat stress during adolescence leads to performance deficits on two separate tasks of spatial working memory. These deficits appear to be dependent on delay duration, with defeated rats making significantly more errors than controls only following a 5 min delay on the delayed win-shift task and only following a 90 s delay on the delayed alternating T-maze task. These results add to previous findings demonstrating that social stress in adolescence also results in deficits in hippocampal-based spatial memory [35-37] and suggest that adolescent stress can have an extensive impact on cognitive function.

Conditioned place preference for task reward (SCM) was similar between groups, suggesting that differences in spatial working memory performance were not due to alterations in the motivation to seek out palatable food. Control rats did demonstrate a significant increase in SCM consumption over time during the conditioning procedure that was not exhibited by previously defeated rats. Given the weak trend for defeated rats to show an increase in consumption over four conditioning sessions (see Figure 1), it is possible that additional sessions would be necessary for this effect to become significant, but further experiments would be required to determine this definitively. However, both total consumption across all conditioning sessions and home-cage consumption did not differ between groups in the current study. Combined with the conditioned place preference results, this further supports the notion that differences in motivation to consume reward did not contribute to memory task performance. Interestingly though, adult rats subjected to adolescent social defeat do show heightened conditioned place preference for amphetamine-paired cues [20]. Therefore, the current finding that similar adolescent social defeat does not augment conditioned place preference for SCM-paired cues may suggest that this social stress during adolescence increases drug but not food reward-related processes. In agreement with the present findings, other studies have demonstrated that male rats subjected to stressors in adolescence do not show later changes in sucrose consumption [49-51], although long term effects on drug reward processes have been more equivocal [20,52,53]. It is known that more chronic social defeat procedures in adulthood can induce anhedonia for both food and drugs [54,55]. However, the adolescent social defeat procedure employed presently may have more in common with episodic as opposed to chronic social defeat paradigms in adulthood, as episodic defeat has been found to increase psychostimulant self-administration without disrupting sucrose preference [55].

Analysis of spatial working memory performance revealed that on the delayed win-shift task, control rats demonstrated an expected increase in errors as the delay was prolonged. This pattern was absent in previously defeated rats, with performance at the 5 min delay similar to that at the 480 min delay. Thus, it appears that in the win-shift task, not only do defeated rats commit more errors compared to controls at the shortest delay used, but their performance also appears to asymptote across subsequent increased delay periods. This is in contrast to the pattern seen on the delayed alternating T-maze, in which defeated rats showed impairments after a 90 s delay, but not after shorter delays of 30 and 60 s. It should be noted that while the delayed alternating T-Maze and delayed win-shift task are similar in respects to their association with mPFC DA and assessment of memory-guided behavior, we are unaware of any studies that have compared the same delay times between these two spatial working memory paradigms. Additional testing would therefore be required to determine definitively the span of delay periods within the same test for which adolescent defeat causes impaired spatial working memory performance.

Because spatial working memory performance on these delayed tasks is known to be dependent upon optimal PFC DA activity [27-33], it is possible that the mPFC DA hypofunction caused by adolescent social defeat [18,22] is contributing to the observed differences in the two spatial working memory tasks. Interestingly, rats experiencing an 85% reduction in mPFC DA content via 6-OHDA lesion exhibit spatial working memory deficits at a 90 s delay on the alternating T-maze, but not at shorter delays of 30 and 60 s [26], similar to the performance of defeated rats in the current study. Additional spatial working memory impairments on the delayed alternating T-maze have been found in rats with deficits in PFC DA activity resulting from either chronic stress [32] or aging [33]. Thus, while other mechanisms cannot be ruled out, disruptions to the developing mPFC DA system during adolescence caused by social defeat stress may contribute to long term deficits in spatial working memory. However, further experiments in which mPFC DA activity in previously defeated rats is restored prior to working memory assessment are required to support such a hypothesis definitively.

Given that increased levels of DA activity are needed to support spatial working memory performance with more prolonged delays [30,31], it might be expected that defeated rats with lower mPFC DA activity would have shown spatial working memory deficits during the 30, 60, and 480 min delay on the delayed win-shift task compared to controls. The lack of differences at these delay intervals between control and previously defeated rats may be due to the role of other brain regions in spatial working memory, specifically at longer delay periods. For example, it is known that an intact connection between the hippocampus and the mPFC is necessary for performance on the delayed win-shift at a 30 min delay, but not during a non-delayed task [34]. A separate study also found that an intact hippocampus became essential for spatial working memory performance at a 5 min delay, but not a 10 s delay [56]. It is therefore possible that the multiple regions involved in spatial working memory performance at longer delays in the delayed win-shift task allowed for compensation in previously defeated rats despite deficits in mPFC DA activity.

An unexpected finding was that despite showing an increased number of spatial working memory errors at 5 min, rats defeated in adolescence required fewer training trials (without a delay) to reach criterion on the delayed win-shift task. It is unlikely that this difference is due to varying levels of motivation for food reward, as both defeated rats and controls showed similar conditioned place preference for and consumption of SCM. One possible explanation for faster criterion acquisition for this task by defeated rats may be an increased preference for novelty. It has been postulated that both alternation behavior on the T-maze and win-shift behavior on the radial arm maze is reminiscent of a rat’s tendency to seek out environmental information in an efficient manner by exploring new places rather than returning to previously visited ones (see reviews [57,58]). Wistar rats selected for naturally high locomotor responses to novelty show an increase in learning performance on a radial arm maze task two days earlier than their low responding peers [59]. Although no differences in locomotion were noted during initial habituation to the apparatus (results not shown), we previously demonstrated increases in adult locomotor behavior in a novel open field and on an elevated plus maze following adolescent social defeat [18,19], as well as upon initial exposure to a CPP apparatus [20]. However, if an increased preference for novelty drove time to reach criterion in the current study, it might be expected that defeated rats would have also required fewer training trials in the alternating T-Maze, which was not the case (although there was a non-significant trend towards faster acquisition in the defeated group). Similar performance between groups during T-Maze training might have been due to the necessary “forced alternation” trials that all rats went under prior to training, which may have served to equalize any potential differences in criterion acquisition in this particular task, negating any effects of increased novelty preference caused by adolescent social defeat that were apparent in the delayed win-shift paradigm. Another potential explanation for faster task acquisition may have to do with differences in learning strategies employed by rats exposed to adolescent social defeat. On maze tasks, rats are known to utilize either a place based/spatial strategy dependent on the hippocampus, or a response based/egocentric strategy dependent on the nucleus accumbens [59]. For win-shift tasks in particular, spatial strategies dependent on the hippocampus are favored to reach initial task acquisition [59,60]. Therefore, while previously defeated rats showed impairment in delayed working memory performance that may be due to deficits in mPFC DA, it is possible that differences in hippocampal monoaminergic activity caused by adolescent defeat [18] promoted enhanced utilization of spatial strategies to acquire the task initially during training.

## Conclusions

These experiments add to an ever growing body of literature in both in humans and animals reporting an adverse effect of stressors during childhood and adolescence on later cognitive function [15,35-37,61-63], which appears to relate to the vulnerability of the developing PFC to stress-induced disruption, particularly by social stress [14,18,21,64]. As stated earlier, executive functions, such as spatial working memory, are known to be impaired in psychiatric disorders associated with social stress during adolescence [8]. Here we have demonstrated that adolescent social defeat directly results in decreased spatial working memory performance in adulthood on two tasks known to be dependent on mPFC DA activity. Given that it is possible to augment mPFC DA activity with various pharmacotherapies, such agents have the potential to reverse the deficits observed in the present study and may have clinical utility for treating victims of adolescent social stress, including bullying. Thus, future experiments will be aimed at utilizing pharmacological manipulations to investigate the relationship between decreases in mPFC DA induced by adolescent social defeat and the observed spatial working memory deficits found in the present study.

## Abbreviations

6-OHDA: 6-hydroxydopamine; ADHD: Attention deficit hyperactivity disorder; CPP: Conditioned place preference; DA: Dopamine; LSD: Least significant difference; mPFC: Medial prefrontal cortex; P: Postnatal day; PFC: Prefrontal cortex; SCM: Sweetened condensed milk.

## Competing interests

The authors declare that they have no competing interests.

## Authors’ contributions

AMN carried out the spatial working memory tasks, helped with the defeat procedure, and drafted the manuscript. LCM carried out the conditioned place preference tests and helped with defeat procedure. GLF oversaw design of the conditioned place preference test and statistical analysis for the entire study, as well as helping to draft the manuscript. MJW conceived the defeat procedure and oversaw design of the entire study and writing of the manuscript. All authors read and approved the final manuscript.
